# 994. Comparison of Lactate, Procalcitonin and a Gene Signature Assay Alone or in Combination to Differentiate Sepsis from Non-infectious Systemic Inflammation in ICU Patients

**DOI:** 10.1093/ofid/ofab466.1188

**Published:** 2021-12-04

**Authors:** Erkan Hassan, Roy Davis, Dayle Sampson, Russell Miller

**Affiliations:** 1 Immunexpress, Clarksville, Maryland; 2 Imtermountain Health cAre, Salt Lake City, Utah

## Abstract

**Background:**

Procalcitonin (PCT) and serum lactate (L) are measures of bacterial infection and tissue hypoxia, respectively, but also used to discern sepsis from infection negative systemic inflammation (INSI). However, improved tools are needed to enhance this differentiation. A previously validated gene signature assay (SeptiCyte RAPID) and its correlated score (SeptiScore (SS)) has been reported to effectively differentiate sepsis from INSI.

**Objective:**

To compare early L, PCT and SS results (alone or in combination) in differentiating sepsis from INSI in adult intensive care unit (ICU) patients (Pt).

**Methods:**

Data from a previously reported, prospective study (8 sites). Inclusion criteria: (i) ICU admission with ≥ 2 signs of systemic inflammatory response syndrome; (ii) Therapeutic antibiotic administration; (iii) external 3-physician clinical review classifying each Pt as sepsis or INSI with ≥ 2 reviewer agreement; (iv) L, PCT & SS values within 24 hrs of ICU admission; (v) Statistical Analysis; (iv) Area under the receiving operator curve (AUROC), 95% confidence intervals (CI) via generalized linear models for: (i) Each parameter alone (L, PCT, SS); (ii) Combinations (L + PCT, L + SS, PCT + SS, All 3); (iii) AUROC discriminated Sepsis from INSI model: (a) < 0.7 Sub-Optimal; (b) 0.7-0.8 Good; (c) > 0.8 Excellent. Comparisons conducted via paired t-test.

**Results:**

222 pts, sepsis=113; INSI=109 Similar demographics between groups (NS). Mean age (SD) = 57.9 (17.1) yrs; 58.1% male). Overall mechanically ventilated 60.8% and hospital mortality 17.1%. AUCROC (95% CI) in Table and Figure; AUCROC of L, PCT or SS alone or in combination

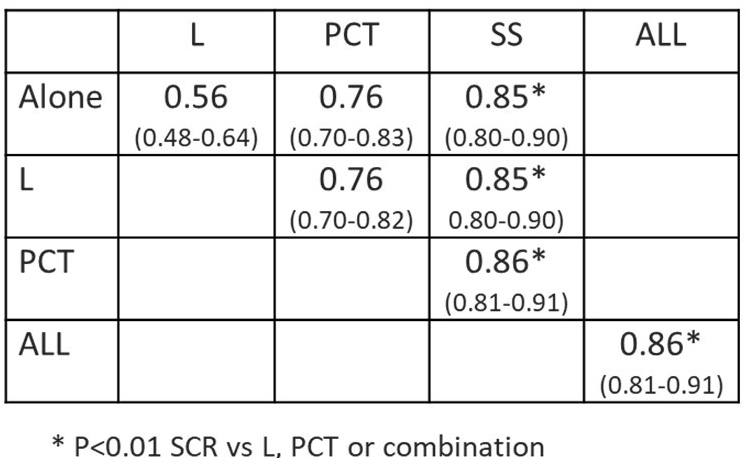

L, PCT, SS Comparison of Sepsis vs INSI

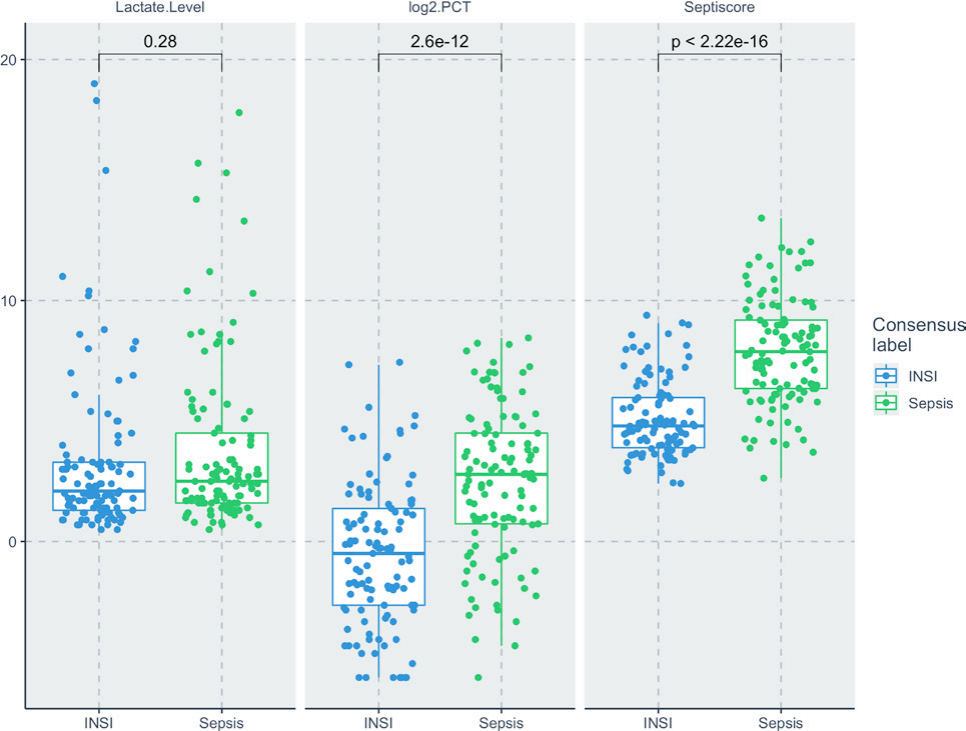

**Conclusion:**

L is sub-optimal in discriminating sepsis from INSI. PCT with or without L was acceptable but not as robust as SS. SS alone or in any combination provided superior and significant discrimination between sepsis and INSI. Incorporation of SS into the clinical assessment process for suspected sepsis pts should be evaluated to determine the impact on early detection and Pt management.

**Disclosures:**

**Erkan Hassan, Pharm.D., FCCM**, **Immunexpress** (Consultant) **Roy Davis, M.D>**, **Immunexpress** (Consultant)**Immunexpress** (Consultant, Shareholder) **Dayle Sampson, Ph.D.**, **Immunexpress** (Employee, Shareholder)

